# LRP1 mediates the IGF-1-induced GLUT1 expression on the cell surface and glucose uptake in Müller glial cells

**DOI:** 10.1038/s41598-021-84090-3

**Published:** 2021-02-26

**Authors:** Virginia Actis Dato, María Cecilia Sánchez, Gustavo Alberto Chiabrando

**Affiliations:** 1 Departamento de Bioquímica Clínica, Facultad de Ciencias Químicas, Universidad Nacional de Córdoba, Haya de la Torre s/n, Ciudad Universitaria, 5000 Córdoba, Argentina; 2 Centro de Investigaciones en Bioquímica Clínica e Inmunología (CIBICI), Consejo Nacional de Investigaciones Científicas y Técnicas (CONICET), Córdoba, Argentina

**Keywords:** Cell biology, Neuroscience

## Abstract

Insulin-like Growth Factor-1 (IGF-1) is involved in the normal development and survival of retinal cells. Low-density lipoprotein Receptor-related Protein-1 (LRP1) plays a key role on the regulation of several membrane proteins, such as the IGF-1 receptor (IGF-1R). In brain astrocytes, LRP1 interact with IGF-1R and the glucose transporter type 1 (GLUT1), regulating the glucose uptake in these cells. Although GLUT1 is expressed in retinal Müller Glial Cells (MGCs), its regulation is not clear yet. Here, we investigated whether IGF-1 modulates GLUT1 traffic to plasma membrane (PM) and glucose uptake, as well as the involvement of LRP1 in this process in the human Müller glial-derived cell line (MIO-M1). We found that IGF-1 produced GLUT1 translocation to the PM, in a time-dependent manner involving the intracellular signaling activation of MAPK/ERK and PI_3_K/Akt pathways, and generated a significant glucose uptake. Moreover, we found a molecular association between LRP1 and GLUT1, which was significantly reduced by IGF-1. Finally, cells treated with specific siRNA for LRP1 showed an impaired GLUT1 expression on PM and decreased glucose uptake induced by IGF-1. We conclude that IGF-1 regulates glucose homeostasis in MGCs involving the expression of LRP1.

## Introduction

The neural retina is one of the most metabolically active tissues in the whole body, which uses glucose to produce energy intermediaries required for daily function^1^. The glucose supply disruption generates a progressive degeneration of the retinal neurons, which results in a severe impairment of the visual function^2^. Müller glial cells (MGCs) are the main macroglia in the retina and they are involved in controlling blood flow, extracellular potassium concentration as well as modulating neuronal activity^3–5^. Several studies in the retina have found a direct relationship between macroglial activation and increased neuronal injury in experimental models associated with altered glucose metabolism, such as in diabetic retinopathy^6–8^. However, how glucose uptake is regulated by MGCs in the retina is not clearly understood.

Insulin-like growth factor-1 (IGF-1), together with cytokines, activates common signaling pathways that are necessary for the reprogramming of MGCs and retinal regeneration upon injury^9^. The IGF-1 signal is intracellularly transduced through its interaction with the cognate receptor (IGF-1R) activating ERK/MAPK and PI_3_K/Akt (PKB) pathways, which potentially leads to cell proliferation or cell differentiation^10^. IGF-1 functions are critical for the protection of nerve cells against neurodegenerative processes^11,12^. Deficiency in the IGF-1 gene in humans was associated with neuronal disorders such as microcephaly, mental retardation, and bilateral sensorineural deafness^13–15^. In the retina, MGCs are influenced by IGF-1 since it can promote migration, proliferation and cell activation, which have implications in retinal neurodegeneration^16,17^. Although IGF-1 is involved in the glucose uptake in central nervous system, its function in neural retina is not clear established yet^18,19^.

The Low-density lipoprotein Receptor-related Protein-1 (LRP1) is an endocytic receptor involved in multiple signaling pathways by its ability to interact and regulate the function of others cell surface receptors in several tissues^20–22^. In this way, it has been demonstrated that LRP1 interacts with insulin receptor (IR) and regulates insulin-induced intracellular signaling and glucose uptake in the brain and cardiomyocytes^23,24^. Glucose enters to cells through transmembrane facilitative glucose transporters (GLUTs 1–5), members of a gene family, which display distinctive tissue distributions, sensitivity to glucose and hormonal regulation^25^. GLUT1 is the main glucose transporter in the retina and facilitates transport of glucose and other substrates^26^. This transporter is expressed in neural retina cells included MGCs and neurons, while GLUT2, GLUT3 and GLUT4 are present in neurons, photoreceptors and ganglion cells^8,27,28^. It was demonstrated that a decrease in GLUT1 into the outer retina of mice reduced glucose levels in this tissue resulting in activation of MGCs and progressive degeneration of the retinal neurons^8,27^. Although GLUT1 is the main glucose transporter in the retina, the regulation of its intracellular traffic and activity in this tissue remains slightly explored.

In brain astrocytes, it was shown that GLUT1 is trafficked to the plasma membrane (PM) through the synergic action of IGF-I and insulin promoting the glucose uptake^19^. Moreover, LPR1 interacts with IGF-1R regulating glucose uptake and GLUT1 traffic to PM also in brain astrocytes^18^. However, whether LRP1 is involved in the glucose uptake through GLUT1 and IGF-1 in retinal MGCs is completely unknown.

In the present work, we evaluated whether IGF-1 induces GLUT1 traffic to PM and glucose uptake, as well as, if LRP1 mediates this process in the human Müller glial-derived cell line, MIO-M1. Here, we found that IGF-1 promoted GLUT1 traffic to PM in a time-dependent manner, involving the intracellular signaling activation of MAPK/ERK and PI_3_K/Akt pathways. This IGF-1-induced GLUT1 expression on the cell surface subsequently generated a significant 2-NBDG uptake. Moreover, IGF-1 reduced the molecular association between LRP1 and GLUT1. Finally, in cells treated with specific siRNA for LRP1 we found an impaired GLUT1 expression on PM and 2-NBDG uptake induced by IGF-1, suggesting that LRP1 plays a key role in the regulation of glucose homeostasis by IGF-1 in MGCs.

## Results

### IGF-1 activates MAPK/ERK and PI_3_K/Akt pathways in MIO-M1 cells

**Figure 1:**
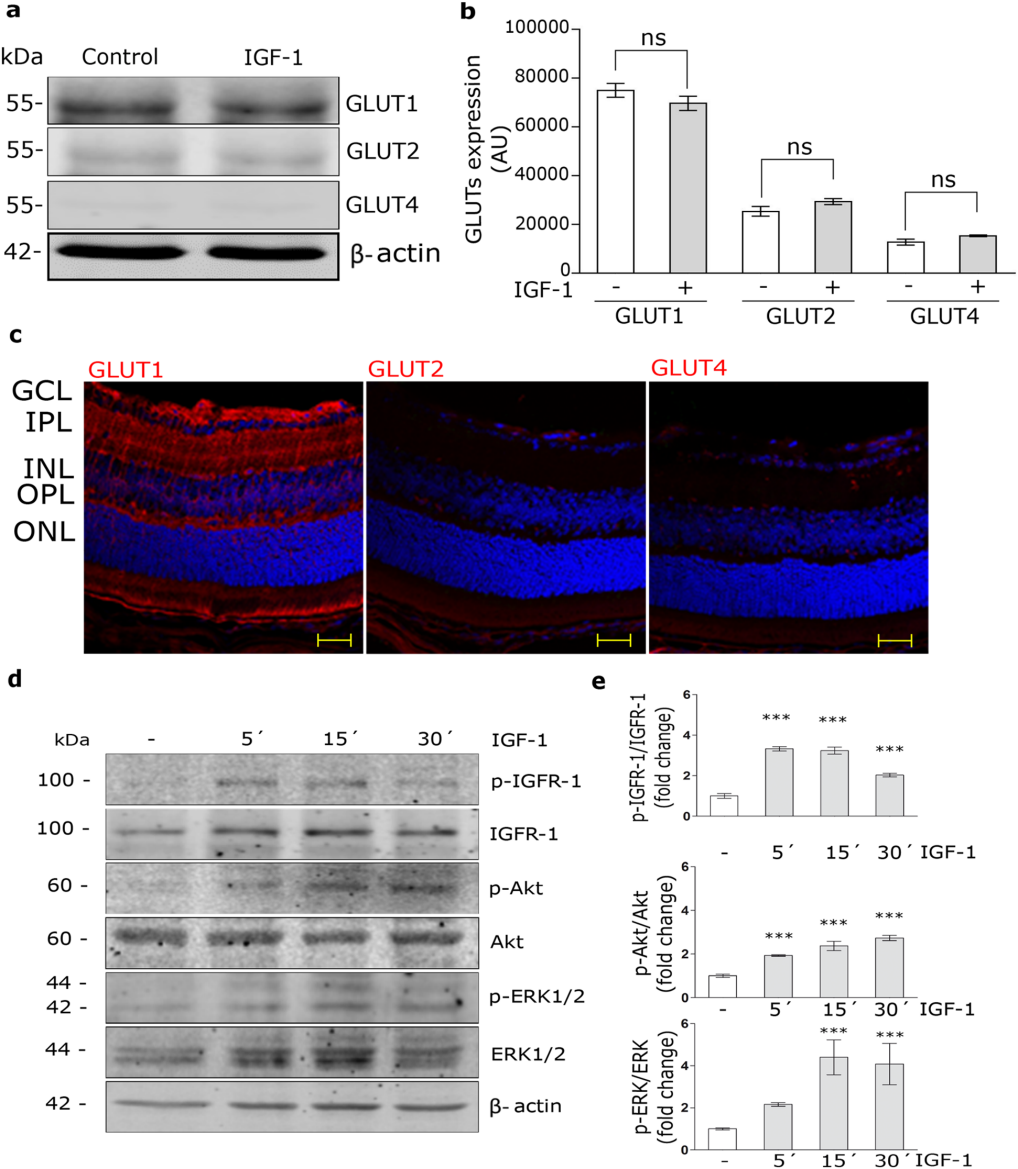
IGF-1 activates MAPK/ERK and PI3K/Akt pathways. (**a**) Western blot assay for the analysis of GLUT1, GLUT2 and GLUT4 expression in MIO-M1 cells treated with IGF-1 10 nM for 60 min. β-actin was used as loading control. (**b**) Densitometric quantification of Western blot data expressed as arbitrary units (AU). Values are expressed as mean ± SEM. ns = non-significant differences. Three independent experiments in duplicate were performed (n = 6). (**c**) Representative immunofluorescence analysis of GLUT1, GLUT2 and GLUT4 (red) in cryosections of mouse retinas (at 26 days of life). Abbreviations: GCL, ganglion cell layer; IPL, inner plexiform layer; INL, inner nuclear layer; OPL, outer plexiform layer; ONL, outer nuclear layer. Scale bar: 25 μm. (n = 3) (**d**) Western blot assay for the analysis of phosphorylated IGF-1R (p-IGF-1R; T1316), Akt (p-Akt; T308) and ERK 1/2 (p-ERK1/2; Thr202/Tyr204) in MIO-M1 cells treated with IGF-1 10 nM for 5–30 min. Total IGF-1R, Akt, ERK1/2 and β-actin were used as loading control. (**e**) Densitometric quantification of Western blot data expressed as fold change respect to non-stimulated control (white bar). Values are expressed as mean ± SEM. ***p < 0.001 versus non-stimulated control. Three independent experiments in duplicate were performed (n = 6).

Although glucose transporters are present in the neural retina, their expression levels in MGCs were not clearly determined. Figure 1a and b show that GLUT1 is expressed in total protein extracts of MIO-M1 cells, as well as, in cryosections of mouse retina with a radial distribution along the entire retina, compatible with the location of the MGCs (Fig. 1c). Although the expressions of GLUT2 and GLUT4 were weakly detected in MIO-M1 cells, these proteins shown a different pattern of distribution compared to GLUT1 in mouse retina (Fig. 1c). Moreover, IGF-1 did not produce significant changes in protein levels of GLUTs for 60 min of stimulus in MIO-M1 cells (Fig. 1a,b).

It is known that the activation of IGF-1R leads to the phosphorylation of MAPK/ERK and PI_3_K/Akt in different tissues^10^. Here, we evaluated the IGF-1 intracellular signaling activation in MIO-M1 cells. Cells were treated with IGF-1 10 nM for 5–30 min. Figure 1c and d show that IGF-1 induced IGF-1R phosphorylation (p-IGF-1R; T1316) at 5–15 min followed by a subsequent activation of Akt (p-Akt; T308) and ERK (p-ERK; Thr202/Tyr204) mainly from 5 and 15 min of stimulus, respectively.

### IGF-1 produces GLUT1 traffic to the PM and glucose uptake in MIO-M1 cells

**Figure 2:**
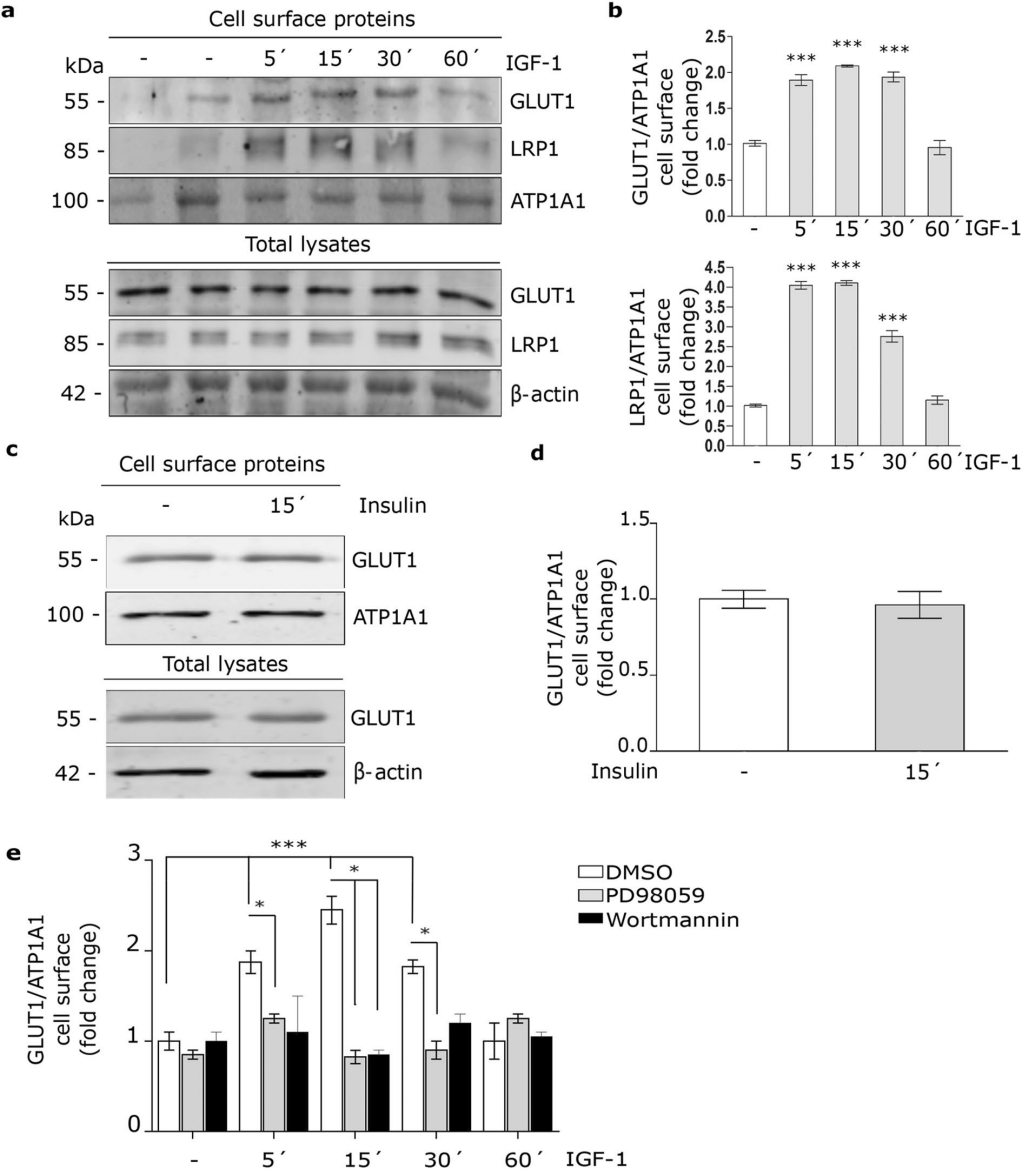
IGF-1 induces GLUT1 traffic to the PM by MAPK/ERK and PI_3_K/Akt signaling activation. (**a**) Biotin-labeling protein assay to measure the expression of GLUT1 and LRP1 in the PM of MIO-M1 cells stimulated with IGF-1 10 nM for 5–60 min. Biotin-labeled proteins were isolated with streptavidin-conjugated beads and then analyzed by Western blot. ATP1A1 and β-actin were used as protein loading controls. Line 1: control without biotin. (**b**) Densitometric quantification of Western blot data for cell surface GLUT1 and LRP1 related to ATP1A1 expressed as fold change respect to non-stimulated control (white bar). Values are expressed as mean ± SEM. ***p < 0.001 versus non-stimulated control. Three independent experiments in duplicate were performed (n = 6). (**c**) Biotin-labeling protein assay to measure expression of GLUT1 in the PM of cells stimulated with Insulin 10 nM for 15 min. Biotin-labeled proteins were isolated with streptavidin-conjugated beads and then analyzed by Western blot. ATP1A1 and β-actin were used as protein loading controls. (**d**) Densitometric quantification of Western blot data for surface GLUT1 related to ATP1A1 expressed as fold change respect to non-stimulated control (white bar). Values are expressed as mean ± SEM. Three independent experiments in duplicate were performed (n = 6). (**e**) Cell surface protein detection assay to measure plasma membrane GLUT1 in cells pretreated with PD98059 (40 μM) or Wortmannin (40 μM) for 30 min and then stimulated with IGF-1 10 nM for 5–60 min. The cell surface level of GLUT1 was analyzed in non-permeabilized cells using anti-GLUT1 antibody as is indicated in detail in Methods. Values are expressed as mean ± SEM. ***p < 0.001 versus non-stimulated control. *p < 0.05 versus indicated conditions. Three independent experiments in duplicate were performed (n = 6).

It has been shown that IGF-1 promotes GLUT1 traffic to PM in brain astrocytes^18^. Here, we evaluated whether IGF-1 may regulates GLUT1 levels on the cell surface. For this, MIO-M1 cells were treated with IGF-1 10 nM for 5–60 min and the GLUT1 level in the PM was analyzed by biotin-labeling cell surface protein assay followed by Western blot. Figure 2a and b show that IGF-1 significantly increased GLUT1 level in the PM respect to control after 5–30 min of stimulus. By contrast, insulin did not produce significant changes in GLUT1 expression on cell surface (Fig. 2c,d), suggesting that the GLUT1 traffic is selectively regulated by IGF-1 in this cell type. In a previous report, we found that insulin induced the LRP1 translocation to PM in MIO-M1 cells^29^. Thus, we evaluated whether IGF-1 may also promote changes of LRP1 on the cell surface. Figure 2a and b show that IGF-1 significantly increased LRP1 expression on PM. Next, we analyzed whether GLUT1 traffic to PM involved IGF-1-induced intracellular signaling activation. Through cell surface protein detection assays we found that IGF-1 effect on GLUT1 traffic to PM was abrogated by both PD98059 (MAPK kinase inhibitor) (40 μM) and Wortmannin (PI_3_K inhibitor) (40 μM) (Fig. 2e), indicating that IGF-1 induces GLUT1 traffic to cell surface through ERK/PI_3_K pathway activation.

**Figure 3:**
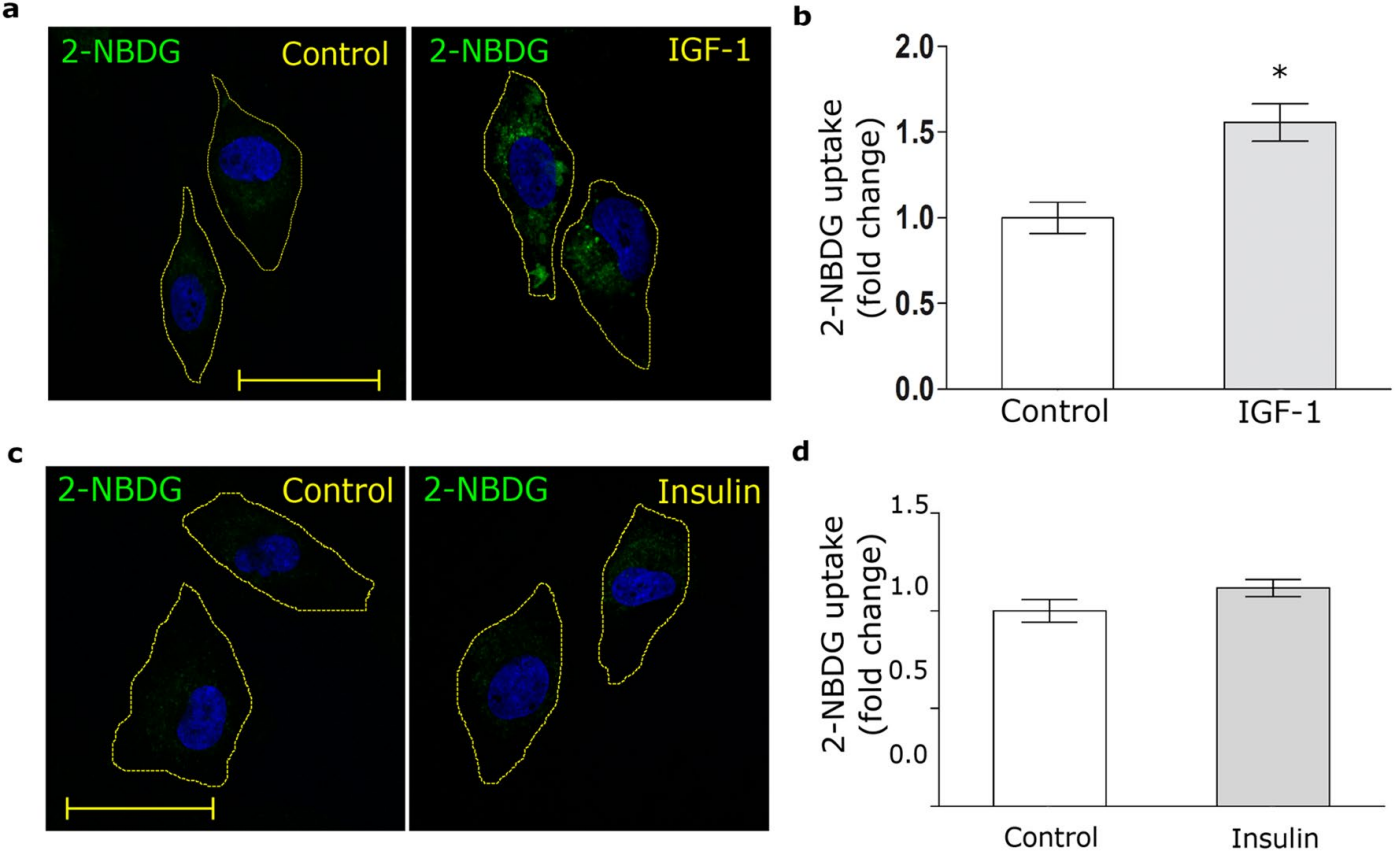
IGF-1 promotes glucose uptake. (**a**,**c**) Confocal microscopy in MIO-M1 cells treated with IGF-1 10 nM (**a**) or Insulin 10 nM (**c**) together with 2-NBDG 80 µM (green) for 30 min. Dotted line represents the cell shape. Images are representative of 20 cells per condition (n = 20). Scale bar = 15 μM. (**b**,**d**) Graph represents mean ± SEM of the fluorescence intensity of 2-NBDG per cell area expressed as fold change. *p < 0.05 versus non-stimulated control (white bar).

Taking into account these results, we evaluated the glucose uptake by MIO-M1 cells. Thus, cells were treated with IGF-1 in the presence of 2-NBDG, a glucose fluorescent analogue, for 30 min. Figure 3a and b show that IGF-1 significantly increased the 2-NBDG uptake. By contrast, insulin did not produce glucose uptake in these cells (Fig. 3c,d).

### IGF-1 reduces the molecular association between LRP1 and GLUT1 in MIO-M1 cells

**Figure 4:**
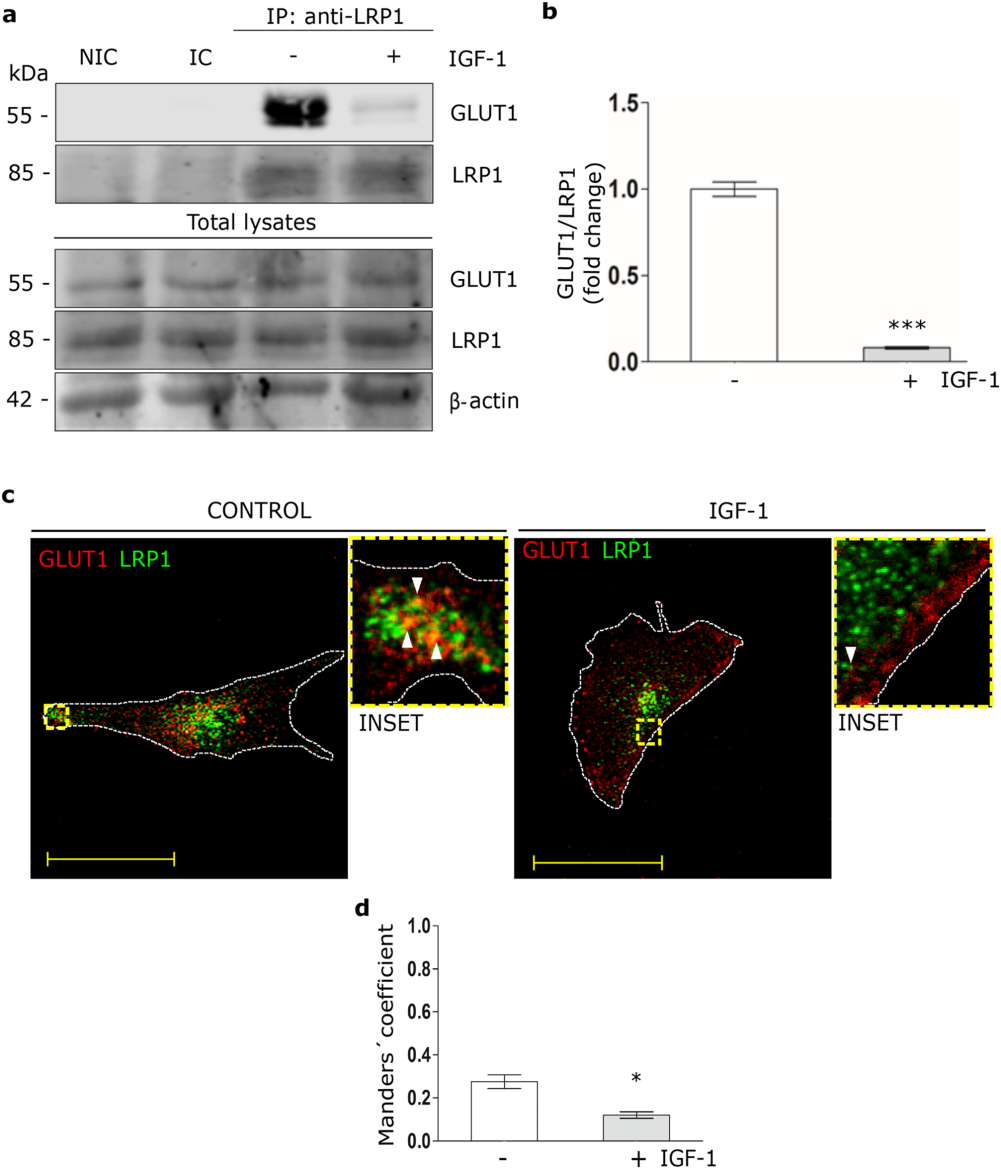
IGF-1 reduces the molecular association between LRP1 and GLUT1. (**a**) Immunoprecipitation assays in MIO-M1 cells treated with IGF-1 10 nM for 30 min. Cell lysates were immunoprecipitated with anti-LRP1 antibody and protein A/G agarose beads as indicated in Methods. Immunoprecipitated LRP1 and GLUT1 are shown on the top panel and total LRP1, GLUT1 and β-actin in cell lysates are shown in the lower panel of the Western blot. NIC: non-immune control. IC: isotype immunoglobulin control. (**b**) Densitometric quantification of relative intensity of GLUT1 bands with respect to LRP1 as a fold change against control (white bar). Values are expressed as mean ± SEM. ***p < 0.001 versus non-stimulated control. Three independent experiments in duplicate were performed (n = 6). (**c**) Confocal microscopy in MIO-M1 cells treated with IGF-1 10 nM for 30 min. Representative images of LRP1 (green) and GLUT1 (red) in immunostained sections. INSET represents magnification 4× of framed regions in dotted lines. White arrowheads indicate colocalization sectors (yellow). Images are representative of 20 cells per condition (n = 20). White dotted line represents the cell shape. Scale bar = 15 μM. (**d**) Quantitative analysis of colocalization between LRP1 and GLUT1 by Manders’ coefficients are expressed as mean ± SD (%) measured in 20 cells per condition from three independent experiments. *p < 0.05 versus non-stimulated control (white bar).

In brain astrocytes it has been shown that LRP1, together with IGF-1R and the scaffolding protein GIPC, are involved to retain GLUT1 inside the cell^18,19^. Taken into account that IGF-1 increases GLUT1 and LRP1 levels on cell surface, we evaluated whether GLUT1 interacts with LRP1 in MIO-M1 cells and if this molecular association is regulated by IGF-1. Thus, cells were treated with IGF-1 10 nM for 30 min. Next, immunoprecipitation procedures were carried out using an anti-LRP1 antibody followed by Western blot assays for GLUT1. Figure 4a and b show that under basal conditions a molecular association is produced between LRP1 and GLUT1, which was significantly reduced by IGF-1. In Fig. 4c and d it is shown that under basal conditions there is a colocalization between LRP1 and GLUT1, which decreased in presence of IGF-1 generating a redistribution of GLUT1 to cell periphery.

### IGF-1-induced GLUT1 traffic to PM and glucose uptake are dependent on LRP1 expression in MIO-M1 cells

**Figure 5:**
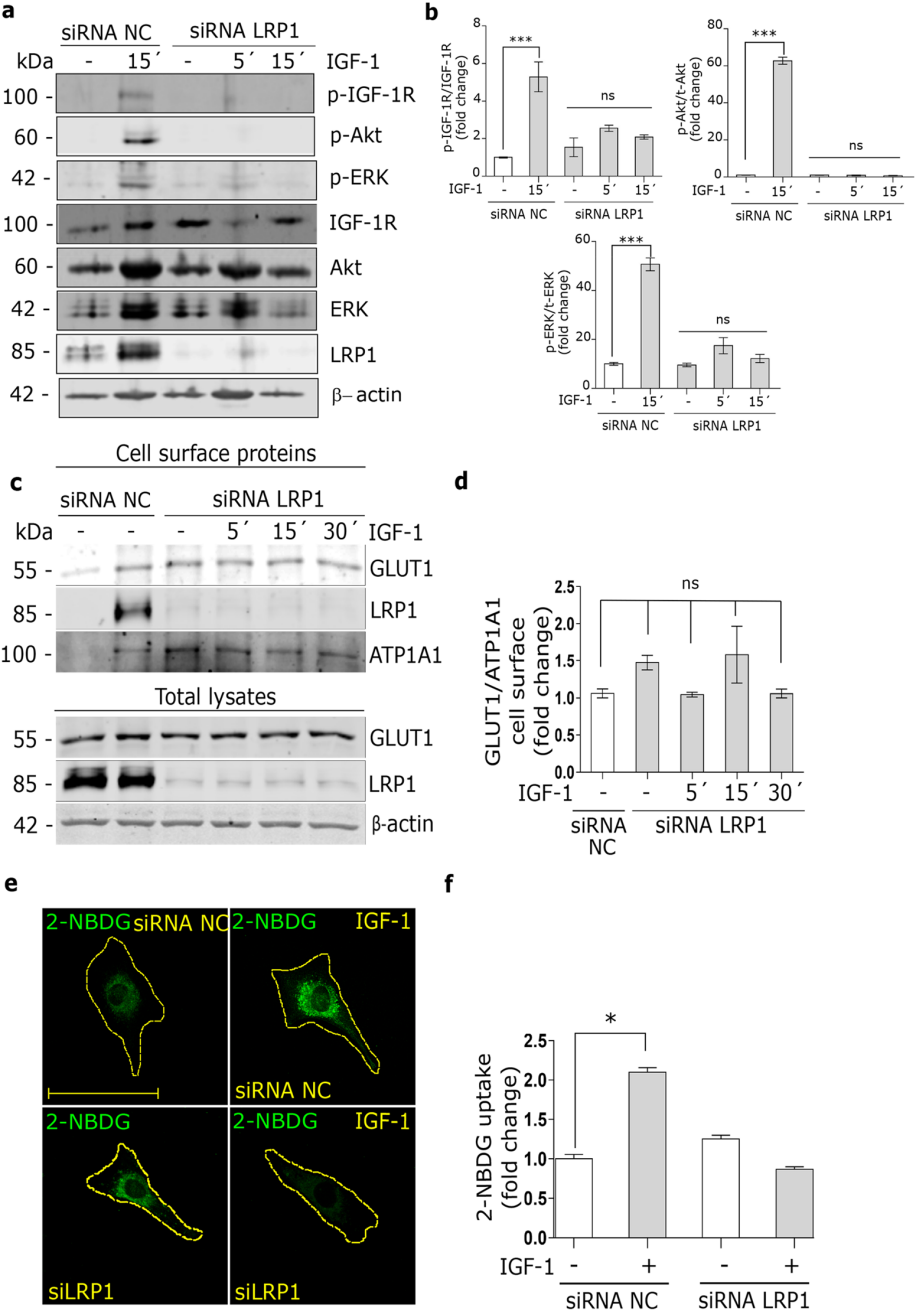
IGF-1-induced GLUT1 traffic to PM and glucose uptake are dependent on LRP1 expression. (**a**) Western blot assay for the analysis of phosphorylated IGF-1R (p-IGF-1R; T1316), Akt (p-Akt; T308) and ERK 1/2 (p- ERK1/2; Thr202/Tyr204) in MIO-M1 cells treated or not with siRNA for LRP1 and then stimulated with IGF-1 10 nM for 5–15 min. Total IGF-1R, Akt, ERK1/2 and β-actin were used as loading control. (**b**) Densitometric quantification of Western blot data expressed as fold change respect to non-stimulated control (white bar). Values are expressed as mean ± SEM. ***p < 0.001 versus non-stimulated control. ns = non-significant differences. Three independent experiments in duplicate were performed (n = 6). (**c**) Biotin-labeling protein assay to measure expression of GLUT1 and LRP1 in the PM of MIO-M1 cells treated or not with siRNA for LRP1 and then stimulated with IGF-1 10 nM for 5–30 min. Biotin-labeled proteins were isolated with streptavidin-conjugated beads and then analyzed by Western blot. ATP1A1 and β-actin were used as protein loading controls. Line 1: control without biotin. (**d**) Densitometric quantification of Western blot data for surface GLUT1 related to ATP1A1 expressed as fold change respect to non-stimulated control (white bar). Values are expressed as mean ± SEM. ns = non-significant differences. Three independent experiments in duplicate were performed (n = 6). (**e**) Confocal microscopy in MIO-M1 cells treated with specific siRNA for LRP1 (siLRP1) and then stimulated with IGF-1 10 nM together with 2-NBDG 80 µM (green) for 30 min. Dotted line represents the cell shape. Images are representative of 20 cells per condition (n = 20). Scale bar = 10 μM. (**f**) Graph represents mean ± SEM of the fluorescence intensity of 2-NBDG per cell area expressed as fold change. *p < 0.05 versus non-stimulated control (white bars).

Previously, we found that LRP1 is required for the insulin intracellular signaling in MIO-M1 cells^22^. Thus, we evaluated whether LRP1 may also be essential for the IGF-1 intracellular signaling. MIO-M1 cells were treated with specific siRNA for LRP1 and then stimulated with IGF-1 10 nM for 5–15 min. Figure 5a and b show that the LRP1 silencing impaired the phosphorylation of IGF-1R, Akt and ERK induced by IGF-1. Next, to evaluate whether LRP1 is also needed to the IGF-1-induced GLUT1 traffic to PM, biotin-labeling cell surface protein assays were carried out in MIO-M1 cells treated with specific siRNA for LRP1 and then stimulated with IGF-1 10 nM for 5–30 min. Figure 5c and d show that the LRP1 silencing abrogated the GLUT1 translocation to the cell surface induced by IGF-1, indicating that LRP1 expression is required for the GLUT1 traffic to PM under IGF-1 stimulus. Taking these data into account, we evaluated whether LRP1 is also involved in the glucose uptake by MIO-M1 cells. LRP1 knockdown cells were treated with IGF-1 10 nM together with 2-NBDG for 30 min. Figure 5e and f show that a decreased LRP1 expression significantly reduced 2- NBDG uptake by IGF-1 respect to control cells. These data suggest that LRP1 mediates the IGF-1-induced GLUT1 traffic to the PM and its subsequent glucose uptake in MIO-M1 cells.

## Discussion

The retina is a highly glycolytic tissue and the glucose entered from systemic circulation is converted in lactate through aerobic glycolysis^30^. Glucose deprivation has been reported to contribute to photoreceptor loss in a number of blinding diseases^8^. On the other hand, high glucose levels in the environmental retinal cells can produce advanced glycation end products and increased oxidative stress reaction, such as occurs in diabetic retinopathy^31^. Although several reports indicated that GLUT1 is the main glucose transporter in different cells of the retina, included retinal pigment epithelium (RPE), photoreceptors and blood-retinal barrier^8,32–35^, the regulation in MGCs is not clearly established yet. IGF-1 is a trophic factor in the retina that plays important roles in normal physiology as well as in pathological states such as retinal neovascularization involved in diabetic retinopathy^36^. IGF-1 also increases the glucose transport into retinal endothelial cells of the blood-retinal barrier, which is mediated by GLUT1^8,36^. In the present work, we found that GLUT1 is expressed in MIO-M1 cells and mouse retina with a location mainly in MGCs. Also GLUT2 and GLUT4 were weakly detected in MIO-M1 cells but they show a different pattern of expression in mouse retina compared to GLUT1. Moreover, the expression levels of these GLUTs were not modified by the presence of IGF-1 in MIO-M1 cells. Finally, we show that IGF-1 induced the GLUT1 traffic to the PM and 2-NBDG uptake, suggesting that this growth factor regulates the glucose homeostasis in MGCs.

Several studies reported that IGF-1 plays a key role in the GLUT1 regulation as glucose transporter in different tissues. In brain, IGF-1 together with insulin promoted the translocation of GLUT1 to the cell surfaces in astrocytes, which was mediated by the activation of MAPK/ERK and protein kinase D (PKD) intracellular signaling pathways^18^. On the other hand, IGF-1 but not insulin induced the GLUT1 traffic to the PM in retinal endothelial cells via the activation of PI_3_K/Akt and protein kinase C (PKC)^36^. In our study, we found through the use of pharmacological inhibitors that IGF-1-induced GLUT1 translocation to the PM is mediated by the activation of MAPK/ERK and PI_3_K/Akt pathways in MIO-M1 cells. Moreover, insulin had not effect in the regulation of GLUT1 translocation to the PM and 2-NBDG uptake in these cells, which is similar to those observed in retinal endothelial cells^36^. This could be related with the differential expression of IGF-1R, insulin receptor (IR) and the hybrid forms (IGF-1R/IR) in MIO-M1 respect to other types of cells. Thus, we suggest that GLUT1 traffic and glucose uptake induced by IGF-1 are selectively regulated in the retina and they may be dependent on the different functions of these cells play into the retinal tissue. In this way, it has been proposed that the regulation of GLUT1 in retinal endothelial cells and RPE are involved with the anabolic and catabolic requirements of photoreceptors^8^, whereas in MGCs the regulation of GLUT1 and glucose uptake induced by IGF-1 may be related with structural and metabolic functions of these retinal cells to preserve neurons and blood vessels influenced by the presence of different extracellular factors^6,37^. Considering that IGF-1 is synthesized by a variety of cell types in the retina other than MGCs^38^, such as microvascular endothelial cells, pericytes, ganglion cells, and RPE^39^, further studies are needed to know if this action of IGF-1 is by autocrine or paracrine way on its cognate receptor expressed by MGCs like occurs in other tissues^40,41^.

In brain astrocytes, it was shown that LPR1 together with IGF-1R are involved in the regulation of GLUT1 traffic to the PM induced by IGF-1^18^. In this way, LRP1 can interact with IGF-1R through its molecular association with the adaptor protein called GIPC (GAIP-interacting protein C) to retain GLUT1 inside the astrocytes^18,19^. However, a putative interaction between LRP1 and GLUT1 was not characterized. In the present study, we found a molecular association between LRP1 and GLUT1 in MIO-M1 cells cultured in the absence of IGF-1, which was in accordance with an increased intracellular retain of GLUT1. By contrast, the presence of IGF-1 produced a significant dissociation of LRP1/GLUT1, which correlated with an enhanced peripheral cell distribution of GLUT1. It is known that LRP1 is predominantly distributed in early endosomes and recycling compartments, which facilitate its intracellular trafficking by endocytosis and endocytic recycling^42^. In previous reports, we observed that LRP1 is stored in small vesicles, LSVs (for LRP1 stored vesicles), which also contain sortilin and GLUT4 ectopically expressed in MIO-M1 cells^22^. These LSVs mediate the intracellular traffic of LRP1 to the PM through the Rab10 and Rab8A activation, inducing regulated exocytosis by insulin^22^ and activated alpha 2-macroglobulin^29^. In the present study, we found that IGF-1 also induces the translocation of LRP1 to the cell surface together with GLUT1. However, further studies are needed to know if the translocation of LRP1 and GLUT1 occur from the same or different subcellular compartments under IGF-1 stimulation.

Finally, in our work we found that LRP1 is required for IGF-1R intracellular signaling and for the translocation of GLUT1 to PM in MIO-M1 cells, since the LRP1 knockdown fully abrogated the phosphorylation of Akt and ERK by IGF-1 and the IGF-1-induced GLUT1 traffic to the cell surface with its subsequent inhibition of 2-NBDG uptake. In this way, it is known that LRP1 is essential for the translocation of GLUT4 to the PM induced by insulin involving at least two mechanisms: (1) an intermolecular interaction between LRP1 and GLUT4 in IRVs, which is required for the GLUT4 traffic to PM^43,44^; and (2) LRP1 acts as a key scaffold protein in the intracellular signaling activation of the IR/PI_3_K/Akt axis, which mediates the GLUT4 translocation to PM^22,24^. In this way, further studies are needed to establish the molecular mechanism by which LRP1 regulates the intracellular traffic of GLUT1 induced by IGF-1.

**Figure 6:**
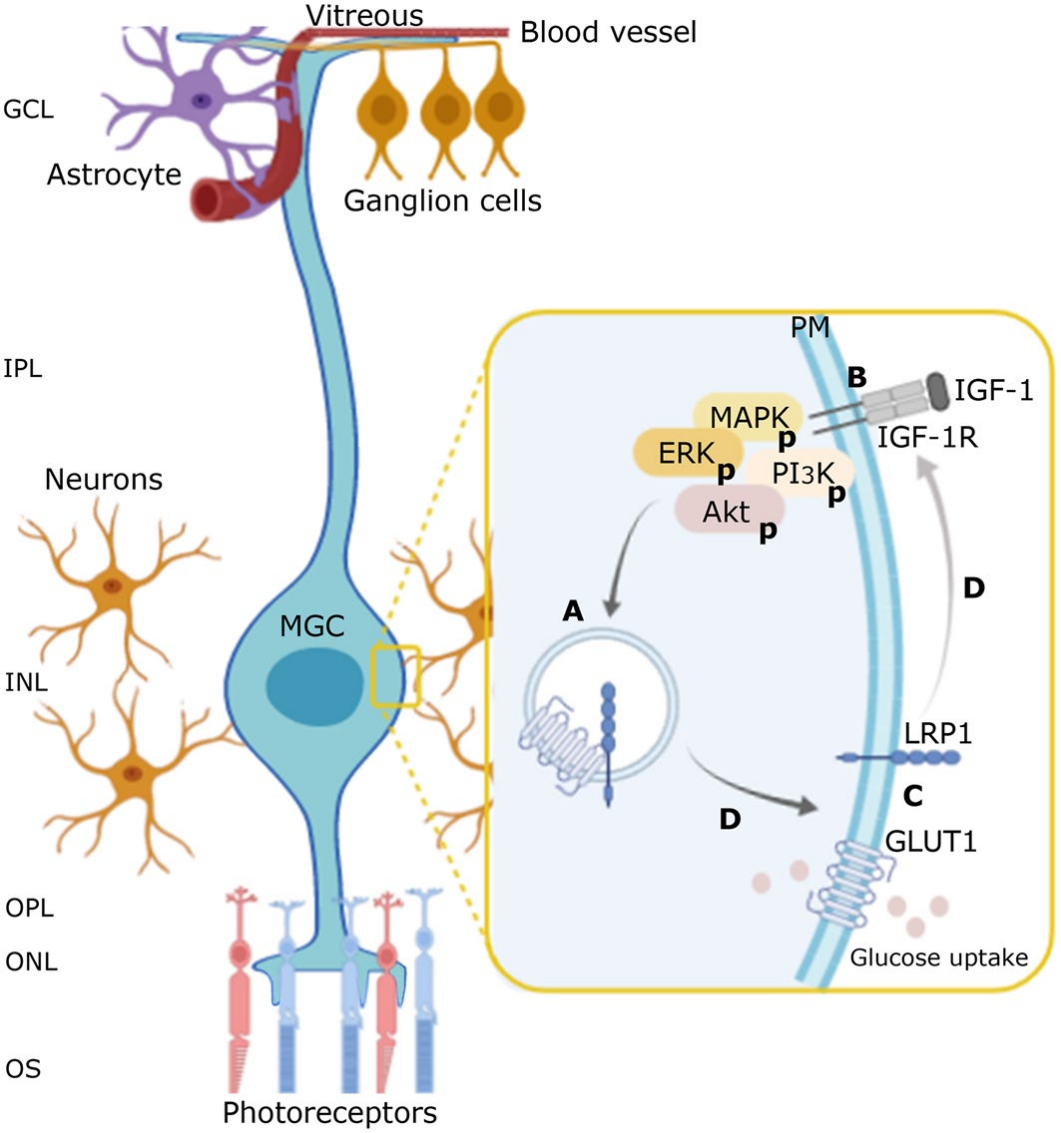
Schematic model of LRP1 mediation in GLUT1 translocation to cell surface and glucose uptake induced by IGF-1. (**A**) Representative image in which, in non-stimulated MIO-M1 cells, LRP1 and GLUT1 are stored in same, but uncharacterized vesicles, since they are molecularly associated through a possible direct interaction or mediated by adaptor proteins. This molecular association would be necessary to retain GLUT1 inside the cells. (**B**) IGF-1 induces MAPK/ERK and PI3K/Akt signaling activation through its cognate receptor (IGF-1R). (**C**) This IGF-1-induced activation promptly leads to the molecular dissociation of LRP1 and GLUT1, promoting the intracellular traffic of both membrane proteins to the PM and glucose uptake. Nevertheless, if both intracellular signaling pathways have different downstream targets on the GLUT1 traffic are still unknown. (**D**) The LRP1 knockdown fully abrogates the IGF-1R intracellular signaling, the GLUT1 translocation and glucose uptake processes. Taken account these considerations, we propose that the LRP1 mediation in the IGF-1-induced glucose control in MIO-M1 cells may be focused at two levels: (1) by regulating the intracellular traffic of GLUT1, and (2) by acting as a scaffold protein for the IGF-1R activation. Ganglion cell layer (GCL), inner plexiform layer (IPL), inner nuclear layer (INL), outer plexiform layer (OPL), outer nuclear layer (ONL), outer segment layer (OS).

The main conclusions of our study are summarized in Fig. 6, providing a novel pathway in the regulation of glucose homeostasis induced by IGF-1, where LRP1 plays a key role in GLUT1 traffic to cell surface and the subsequent glucose uptake in MIO-M1 cells. However, further *in vivo* experiments will be needed to evaluate if this mechanism may control other retinal cells that are metabolically dependent of MGCs, such as neurons and endothelial cells, which could have clinical and therapeutic connotations in neurodegenerative diseases of the retina.

## Methods

### Cell cultures and reagents

MIO-M1 cells, a spontaneously immortalized human Müller cell line (Moorfields/Institute of Ophthalmology-Müller 1), was provided by Dr G. Astrid Limb (University College London, Institute of Ophthalmology and Moorfields Eye Hospital, London, UK) and maintained in DMEM-high glucose (4.5 mg/ml) stabilized with 2 mM l-glutamine (GlutaMAX; Invitrogen, Buenos Aires, Argentina) and supplemented with 110 mg/ml sodium pyruvate, 10% (v/v) fetal bovine serum (FBS) (Invitrogen, Buenos Aires, Argentina) and 100 U/ml penicillin/streptomycin (Invitrogen, Buenos Aires, Argentina) at 37 °C with 5% CO_2_^22,45^. Human IGF-1, Wortmannin and PD98059 were from Sigma-Aldrich (St. Louis, MO). Rabbit anti-pIGF-1R (#28897, T1316), rabbit anti-Akt (#9272), rabbit anti-pAkt (#9275S, T308), rabbit anti-ERK1/2 (#4695) and rabbit anti-pERK (#9101, Thr202/Tyr204) antibodies were from Cell Signaling Technology (Beverly, MA). Mouse monoclonal anti-β-actin (#A2228) antibody was from Sigma–Aldrich (St. Louis, MO). Rabbit anti-LRP1 (#ab92544), rabbit anti-IGF-1R (#ab182408) and mouse monoclonal anti-GLUT1 (#ab40084), rabbit anti-GLUT2 (#ab95256) and rabbit anti-GLUT4 (#ab654) were from Abcam (Cambridge, MA). Mouse monoclonal anti-APT1A1 (#M7-PB-E9) was from ThermoFisher Scientific (Rockford, IL). Several Alexa Fluor conjugated secondary antibodies (goat anti-rabbit IgG- and anti-mouse IgG-Alexa Fluor 594 or 488; Invitrogen, Buenos Aires, Argentina) were used for immunofluorescence assays (antibody dilution: 1/800)^22,29^.

### Transfection procedure for the silencing LRP1

MIO-M1 cells (4 × 10^5^ cells/well) were cultured in 6-well plates and transiently transfected with 5 pmol/well of siRNA-LRP1 (#s8280; Ambion, Austin, TX) for 48 h, using Lipofectamine RNAiMAX reagent (Invitrogen) and Opti-MEM 1× (Gibco, Thermo Fischer Scientific, Buenos Aires, Argentina). As control, the silence select negative control siRNA (#4390846; Ambion) was used^22^.

### Western blot assays

MIO-M1 cell protein extracts were prepared using RIPA buffer (50 mM Tris–HCl pH 8.0, 150 mM NaCl, 1% Triton X-100, 0.5% sodium deoxycholate, 0.1% SDS, 1 mM PMSF, 10 mM sodium ortho-vanadate, and protease inhibitor cocktails (Sigma-Aldrich, St. Louis, MO, USA)). Forty micrograms of cell protein extracts were diluted in sample buffer 5× with DTT (dithiothreitol) and then heated for 5 min at 95 °C. Electrophoresis on 10% SDS–polyacrylamide gels^46^ was applied and proteins were electrotransferred to nitrocellulose membrane^47^ (GE Healthcare Life Science, Amsterdam, The Netherlands). Nonspecific binding was blocked with 5% non-fat dry milk in a Tris–HCl saline buffer containing 0.01% Tween 20 (TBS-T) for 60 min at room temperature. The membranes were incubated overnight at 4 °C with diluted primary antibodies and secondary antibodies raised in goat anti-mouse IgG IRDye 680CW and goat anti-rabbit IgG IRDye 800CW (LI-COR Biosciences, Lincoln, NE) diluted 1/10,000 for 1 h at room temperature. The specific bands were revealed using Odyssey CLx near-infrared fluorescence imaging system (LI-COR Biosciences).

### Biotin-labeling cell surface protein assay

MIO-M1 cells were treated with IGF-1 10 nM for different times (5–60 min). A biotin-labeling protein assay (EZ-Link Sulfo-NHS-SS-Biotin [cat: 21331]), Thermo Scientific, Rockford, IL) was used to measure the level of GLUT1 and LRP1 at the cell surface as previously described^23^. Briefly, streptavidin-conjugated agarose beads (Pierce Streptavidin Agarose [cat: 20353], Thermo Scientific) were used to pull down the biotinylated proteins for 2 h at room temperature. The biotinylated and total proteins were treated for Western blot using rabbit anti-LRP1 (1/10,000), rabbit anti-GLUT1 (1/1000), mouse monoclonal anti-ATP1A1 (1/1500), or mouse monoclonal anti-β-actin (1/5,000) monoclonal antibodies, overnight at 4 °C, and secondary antibodies (goat anti-mouse IgG IRDye 680CW and goat anti-rabbit IgG IRDye 800CW; LI-COR Biosciences) diluted 1/10,000 for 1 h at room temperature. The specific bands were revealed using Odyssey CLx and were quantified by Image Studio Software (LI-COR Biosciences).

### Cell surface protein detection assay

MIO-M1 cells were pre-incubated 30 min with 40 µM wortmannin or PD98059 and then were treated with IGF-1 10 nM for different times (5–60 min). Cells were washed with cold PBS, fixed with 4% (v/v) paraformaldehyde (PFA) and blocked with 5% (v/v) horse serum for 30 min on ice^22^. Cells were incubated with anti-GLUT1 (1/1,000) and anti-ATP1A1 (1/1,500) antibodies for 1 h on ice, and then treated with goat anti-mouse IgG IRDye 800CW (LI-COR Biosciences) secondary antibody (1/10,000) for 1 h on ice. Fluorescence was measured by the Odyssey CLx and quantified by Image Studio Software.

### Confocal microscopy

The procedures were followed as previously described^29,42^. Briefly, MIO-M1 cells were cultured as above on cover glass. After 30 min of IGF-1 stimulus the cells were washed with PBS 1×, fixed with 4% PFA, quenched with 50 mM NH_4_Cl, permeabilized for 30 min with 0.5% (*v*/*v*) saponin, blocked with 2% bovine serum albumin (BSA) and incubated with primary antibodies mouse anti-GLUT1 (1/100) and rabbit anti-LRP1 (1/200) for 1 h, and revealed with a secondary antibody conjugated with Alexa Fluor 594 or 488 (1/800) and Hoechst colorant (1/2000) for 1 h. Finally, the cells were mounted on glass slides with Mowiol 4–88 reagent from Calbiochem (Merck KGaA, Darmstadt, Germany). For co-localization analyses, fluorescent images were obtained with an Olympus FluoView FV1200 confocal laser scanning biological microscope (Olympus, NY). Whole cells were scanned and optical sections were obtained in 0.25-μm steps perpendicular to the *z*-axis, with images being processed using the FV10-ASW Viewer 3.1 (Olympus) and quantified by ImageJ software (NIH, Bethesda, Maryland)^48^.

### Retinal cryosection immunostaining

The procedure was followed as previously described^16^. Briefly, the mice were euthanized at 26 days of life and their eyes were enucleated and fixed with 4% PFA overnight. Then, they were incubated in 10% (2 h), 20% (2 h), and 30% sucrose/PBS overnight at 4 °C, and placed on plastic tubs with a small amount of optimal cutting temperature (OCT) compound (Crioplast, Biopack, Buenos Aires, Argentina). Serial sections (of 8-μm thick) were obtained by using a cryostat (HM 325, Thermo Scientific). Finally, cryosection were incubated with anti-GLUT1 (1/100) anti-GLUT2 (1/100) and anti-GLUT4 (1/100) overnight at 4 °C and with a secondary antibody conjugated with Alexa Fluor 594 (1/800) and Hoechst colorant (1/2000) for 1 h. Samples were mounted with Mowiol 4–88 reagent. Fluorescent images were obtained with an Olympus FluoView FV1200 confocal laser scanning biological microscope (Olympus, NY) and quantified by ImageJ software (NIH, Bethesda, Maryland).

### Immunoprecipitation (IP) assay

MIO-M1 cells were treated with IGF-1 10 nM for 30 min. Cell lysates were obtained using RIPA buffer and then incubated for 2 h at 4 °C with anti-LRP1 antibody or non-immune IgG as control (2 μg/200 μg of total proteins) following the procedure previously described^49^. Briefly, the samples were incubated overnight at 4 °C with protein A-conjugated agarose beads (sc-2001; Santa Cruz Biotechnology, Santa Cruz, CA). The proteins were treated for Western blot using anti-GLUT1 (1/1000) and anti-LRP1 (1/10,000) antibodies. Secondary antibodies (goat anti-mouse IgG IRDye 680CW and goat anti-rabbit IgG IRDye 800CW) were used (antibody dilution: 1/10.000) for 1 h at room temperature. The specific bands were revealed using Odyssey CLx and quantified by Image Studio Software. β-actin was used as loading control of total protein extracts.

### 2-NBDG uptake assay

MIO-M1 cells were stimulated with IGF-1 10 nM for 30 min together with 80 μΜ of 2-Deoxy-2-[(7-nitro-2,1,3-benzoxadiazol-4-yl) amino]-d-glucose (2-NBDG solution; Sigma-Aldrich, St. Louis, MO)^50^. After stimulus, cells were treated as was previously described^23^. Cells were mounted on glass slides with Mowiol 4–88 reagent. Fluorescent images were obtained with an Olympus FluoView FV1200 confocal microscope. Cells were scanned in optical Sections (0.25-μm steps perpendicular to the *z*-axis) and images were processed using the FV10-ASW Viewer 3.1 (Olympus). The total fluorescence in the whole cell area was quantified by ImageJ software.

### Statistical treatment of data

For confocal microscopy, at least 50 cells/condition were analyzed and the colocalization levels was quantified by JACoP plug-in from ImageJ^48^. The averages of the vesicle percentages containing both proteins were calculated using the Manders’ coefficients and compared by the Student’s t-test. For other assays, the data was expressed as the mean ± SEM and comparisons were performed using a paired *t*-test or one-way ANOVA followed by Dunnett’s post-hoc analysis (GraphPad Prism 5.0, San Diego, CA). Values significance (*p* < 0.05).

## Supplementary Information

Supplementary Information 1.

## Abbreviations

Akt or PKB: Protein kinase B
p-Akt: Phospho-Akt
ERK: Extracellular signal-regulated kinase
p-ERK: Phospho-ERK
GLUT1: Glucose transporter type 1
IGF-1: Insulin-like growth factor-1
MGCs: Müller glial cells
LRP1: Low-density lipoprotein receptor-related protein-1
PI_3_K: Phosphatidylinositol-4,5-bisphosphate 3-kinase
siRNA: Small interference RNA
2-NBDG: (2-(N-(7-Nitrobenz-2-oxa-1,3-diazol-4-yl) Amino)-2-Deoxyglucose
