# Supplementary material for: LRP1 mediates the IGF-1-induced GLUT1 expression on the cell surface and glucose uptake in Müller glial cells
Source: Sci Rep. 2021 Feb 26;11:4742. doi: 10.1038/s41598-021-84090-3 (PMC7910306; doi:10.1038/s41598-021-84090-3)
Supplement: Supplementary file 1 — Supplementary Information 1. [file 41598_2021_84090_MOESM1_ESM.pdf]

Supplementary Information

LRP1 mediates the IGF-1-induced GLUT1 expression on the cell surface and glucose uptake in Müller glial cells.

Virginia Actis Dato, María C. Sánchez and Gustavo A. Chiabrando*.

Figure 1 A.

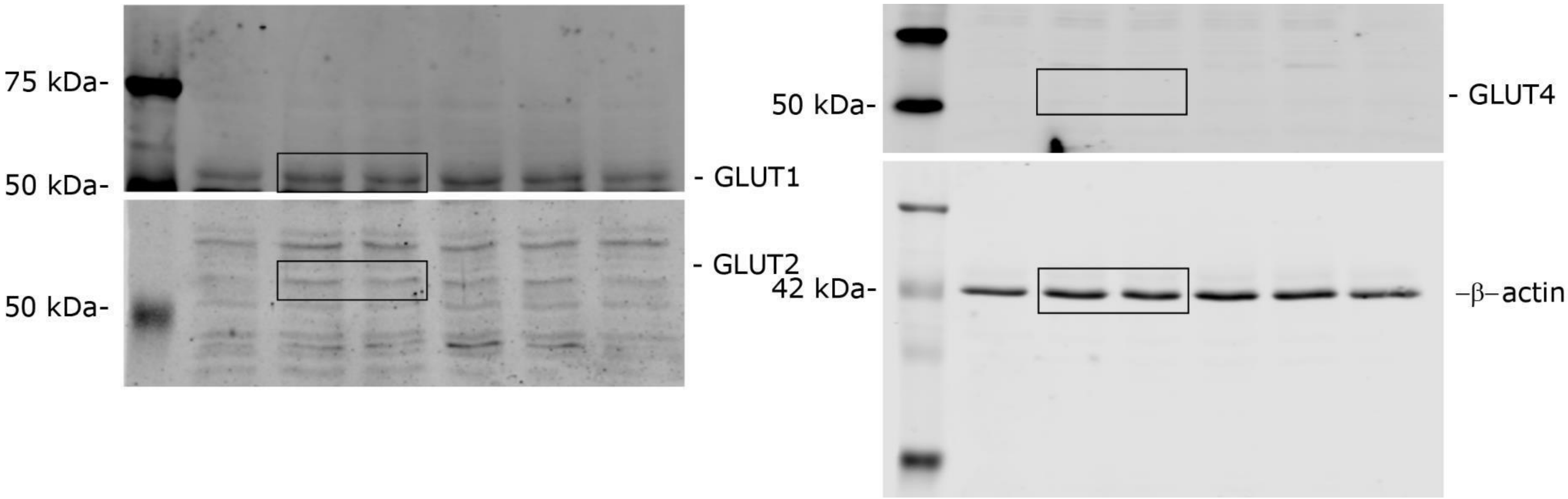

Note: blots for GLUT1, GLUT2 and GLUT4 were acquired in specific regions selected according to molecular weights directly from Odyssey CLx to eliminate non-specific fluorescence or other previously detected proteins and optimize the detection of the proteins of interest. Blots were scanned in black and white.

Figure 1 C.

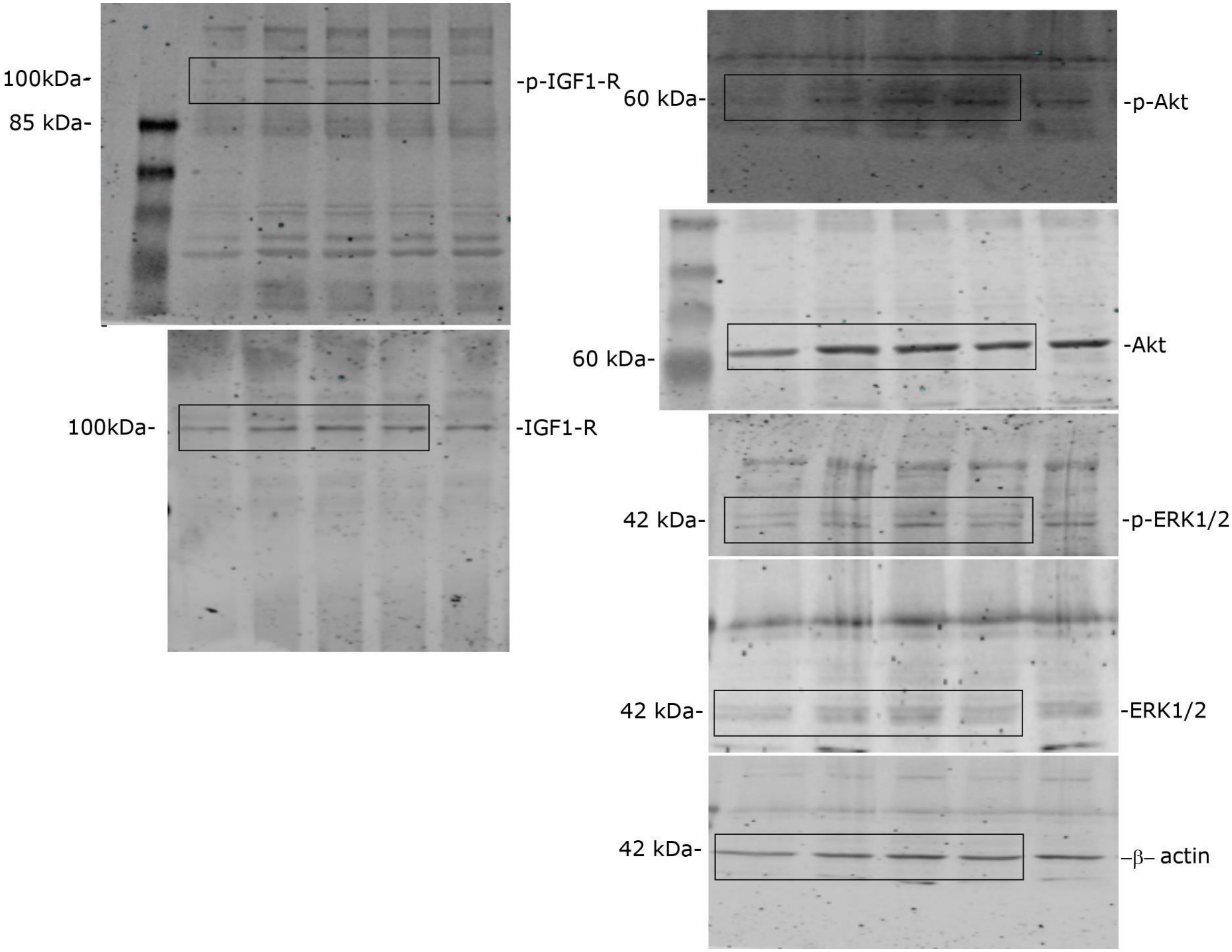

Note: blot for IGF1-R was scanned in specific regions selected according to molecular weights from the blot of p-IGF1-R. Blots for p-Akt, p-ERK1/2, ERK1/2 and β-actin were scanned in specific regions selected according to molecular weights from the blot of Akt. These acquisitions were made in this way to eliminate the fluorescence of the markers that interferes in the detection of the proteins of interest directly from Odyssey CLx. Blots were scanned in black and white.

Figure 2 A.

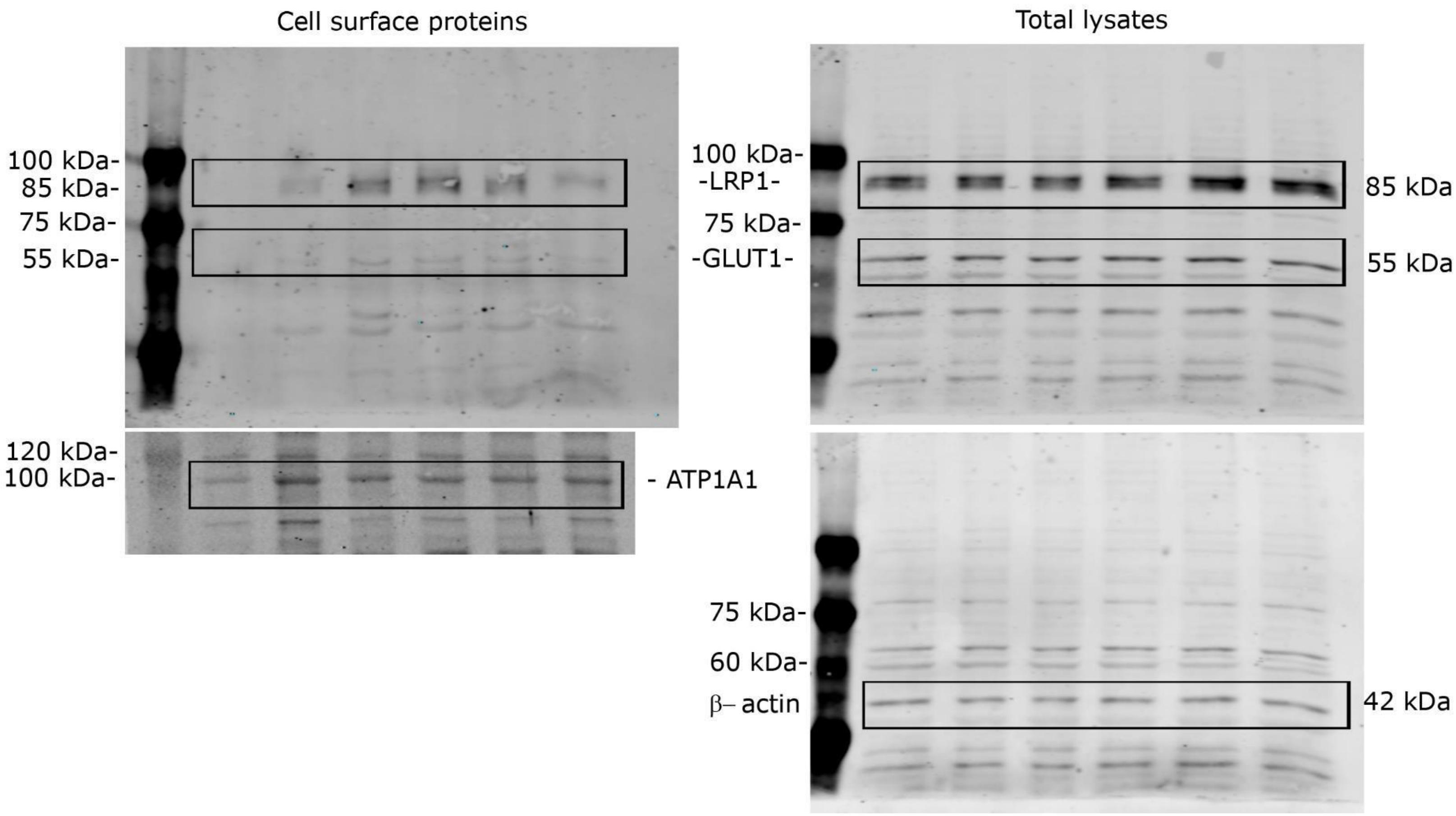

Note: blot for ATP1A1 was acquired in specific regions selected according to molecular weights directly from Odyssey CLx to eliminate non-specific fluorescence or other previously detected proteins and optimize the detection of ATP1A1. Blots were scanned in black and white.

Figure 2 C.

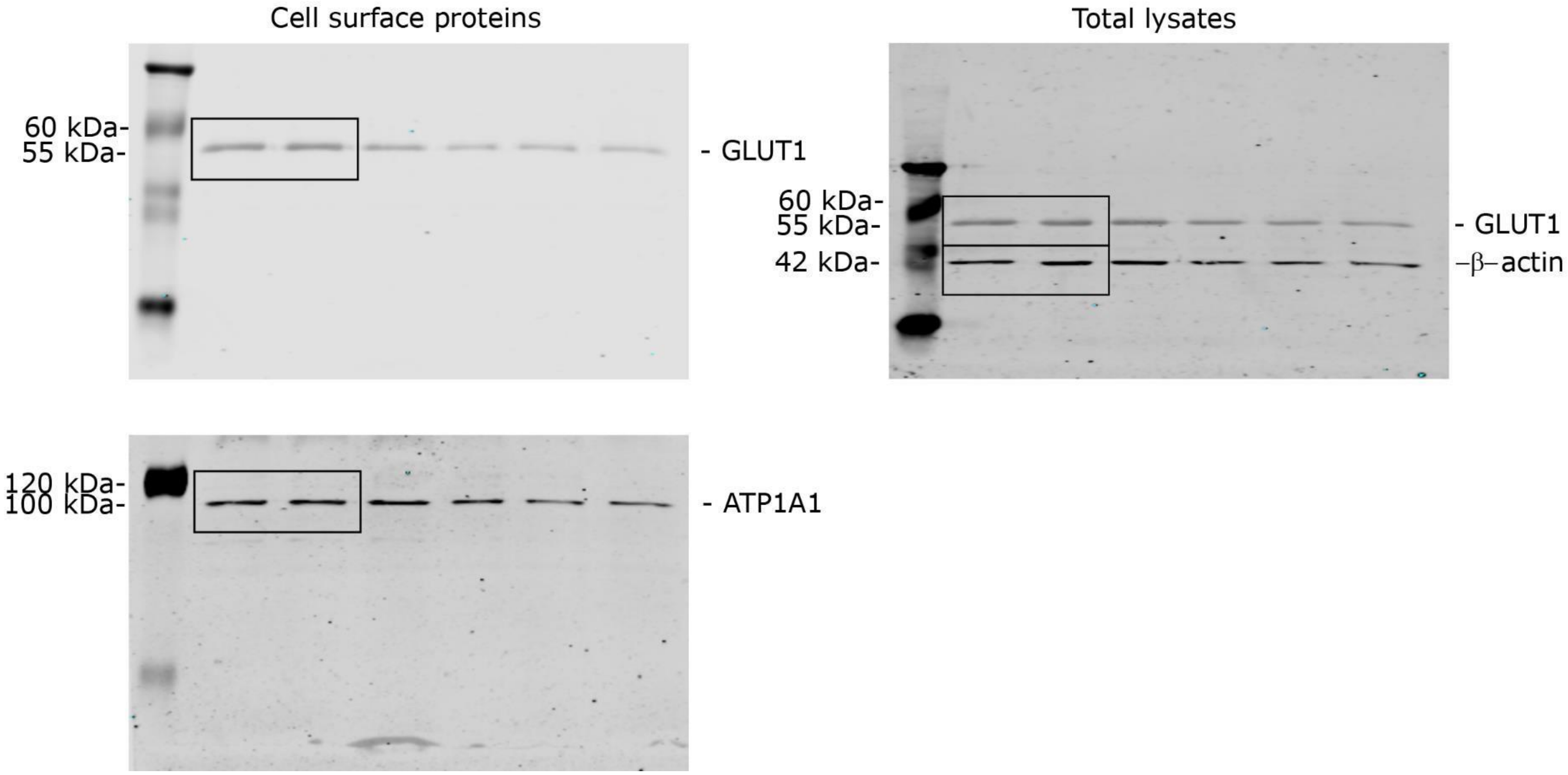

Note: Blots were scanned in black and white.

Figure 4 A.

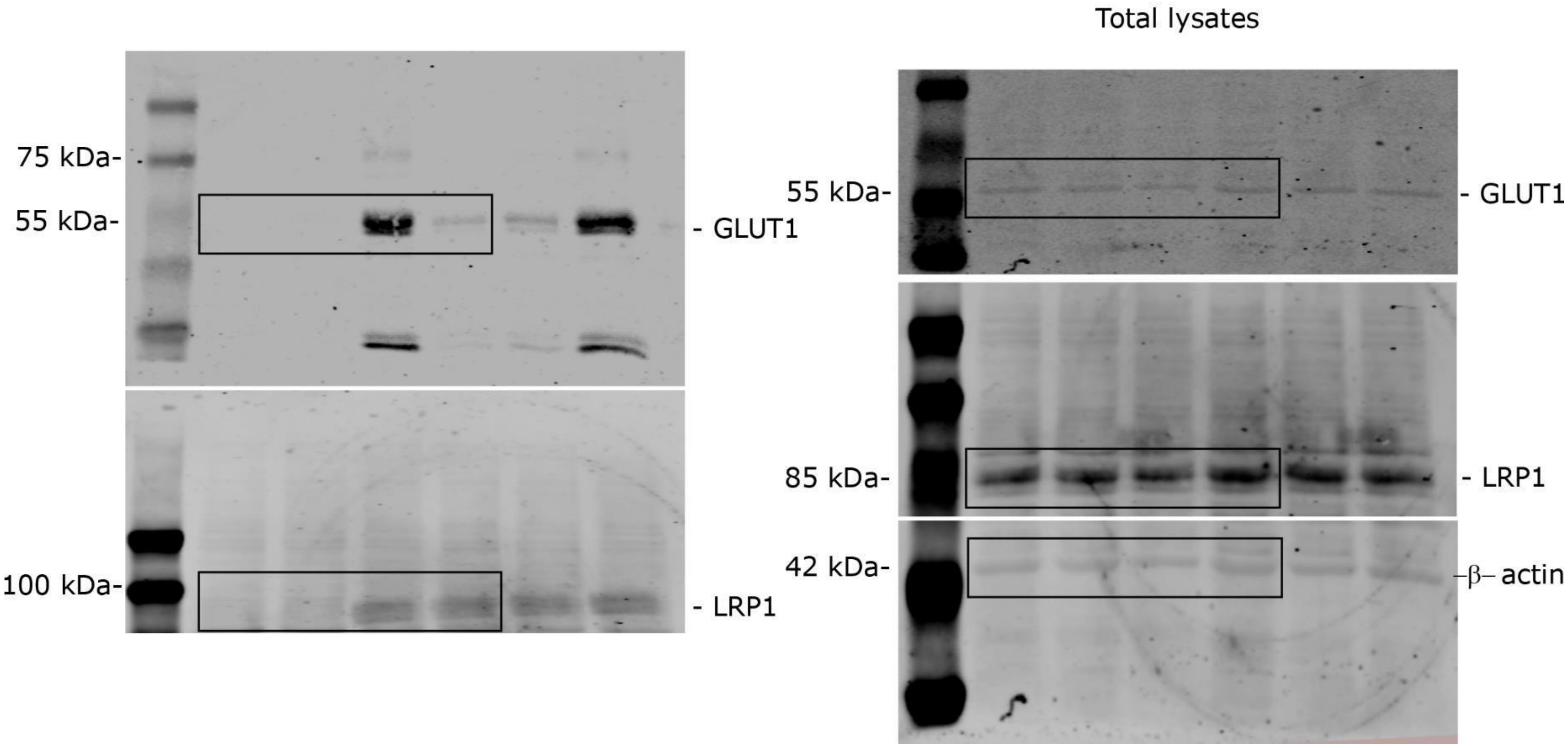

Note: blots for LRP1 (IP), GLUT1(total lysates) and β-actin (total lysates) were acquired in specific regions selected according to molecular weights directly from Odyssey CLx to eliminate non-specific fluorescence or other previously detected proteins and optimize the detection of proteins of interest. Blots were scanned in black and white.

Figure 5 A.

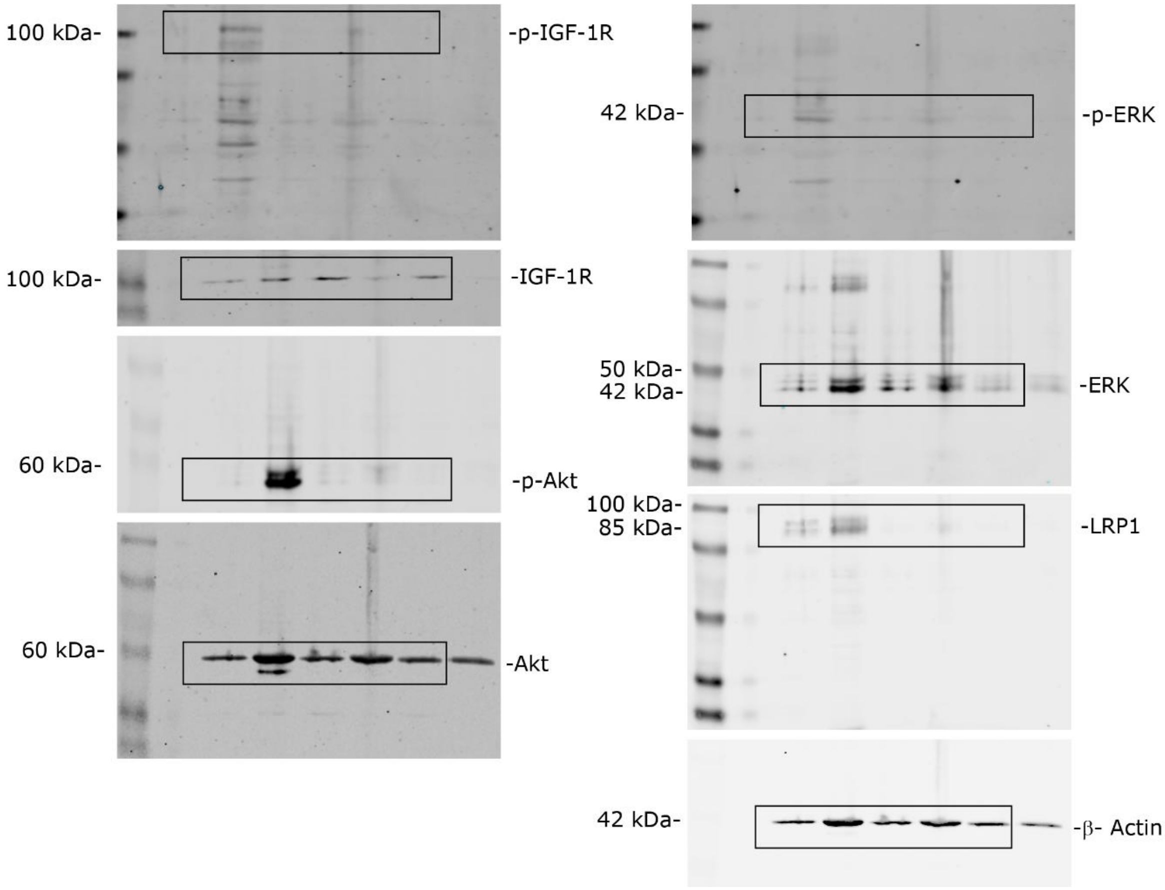

Note: blots for IGF-1R and β-actin were acquired in specific regions selected according to molecular weights directly from Odyssey CLx to eliminate non-specific fluorescence or other previously detected proteins and optimize the detection of proteins of interest. Blots were scanned in black and white.

Figure 5 C.

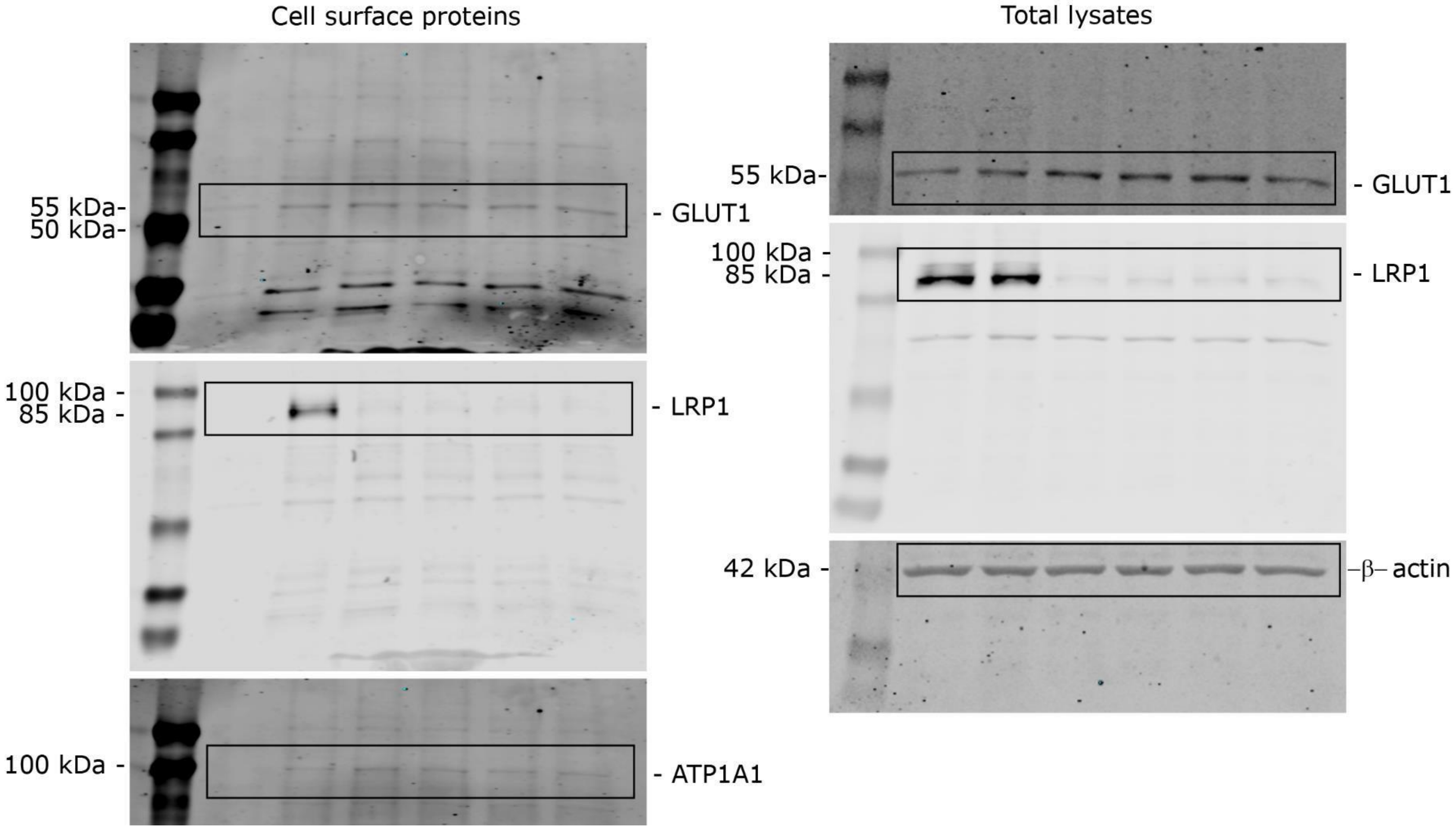

Note: blots for ATP1A1 (cell surface), GLUT1(total lysates) and β-actin (total lysates) were acquired in specific regions selected according to molecular weights directly from Odyssey CLx to eliminate non-specific fluorescence or other previously detected proteins and optimize the detection of proteins of interest. Blots were scanned in black and white.
